# Distribution and Appearance of Ki-67, IL-1α, IL-10, and PGP 9.5 in Reinke’s Oedema-Affected Larynx Tissue Compared with Control Tissue

**DOI:** 10.3390/life11121379

**Published:** 2021-12-10

**Authors:** Vita Konopecka, Mara Pilmane, Dins Sumerags, Gunta Sumeraga

**Affiliations:** 1 Institute of Anatomy and Anthropology, Riga Stradins University, LV-1007 Riga, Latvia; mara.pilmane@rsu.lv; 2 Cesu Clinic, LV-1010 Riga, Latvia; dins.sumerags@cesuklinika.lv; 3 Pauls Stradins Clinical University Hospital, LV-1002 Riga, Latvia; gunta.sumeraga@rsu.lv

**Keywords:** Reinke’s oedema, IL-10, IL-1α, Ki-67, PGP 9.5, larynx

## Abstract

Smoking, laryngopharyngeal reflux, and vocal fold abuse can promote the development of Reinke’s oedema, leading to vocal fold dysfunction and injury. The aim of the work was to investigate the appearance and distribution of proliferation marker Ki-67 (Ki-67), interleukin 10 (IL-10), interleukin 1 alpha (IL-1α), and protein gene peptide 9.5 (PGP 9.5) in Reinke’s oedema-affected larynx tissue. Methods: A routine histological and immunohistochemical Reinke’s oedema and control group patient analysis was conducted. We used the biotin–streptavidin biochemical method to detect Ki-67, IL-10, IL-1α, and PGP 9.5 The semiquantitative grading method was used to evaluate immunoreactive cells’ appearance and local distribution. A Mann–Whitney U test and Spearman’s rank coefficient were performed. Results: A low positive correlation between IL-1α epithelial and subepithelial immunoreactive cells in the patient group was found. Mann–Whitney U tests revealed significant patient and control group immunoreactive marker differences. All examined markers showed a higher number of immunoreactive structures in the patient group. Conclusions: Intensive proliferation of the surface epithelium was observed in patient tissues. The notable increase in IL-10 positive structures indicates the dominant anti-inflammatory tissue response. An increased number of IL-1α structures in the larynx epithelium and subepithelium in the patient group is linked to inflammation, proliferation, and tissue remodelling. The PGP 9.5 expression increase is involved in the morphopathogenesis of Reinke’s oedema.

## 1. Introduction

Reinke’s oedema (RE) is the benign diffuse swelling of the vocal folds, also known as various polypoid degeneration or smokers’ polyps. These lesions can be unilateral as well as bilateral [1]. The general population prevalence of Reinke’s oedema is 3.47/1000 [2]. Changes appear in the superficial lamina propria (SLP), also called Reinke’s space, which has an important functional role in vocal fold vibrations as well as voice production [3].

Reinke’s space consists of regularly interrelated parallel connective tissue fibres. An accumulation of the oedematous transudate in Reinke’s space can lead to varying levels of dysfunction of the vocal folds. During the phonation process, the oedematous transudate is in motion. The overexpression of the glottic wave created by Reinke’s oedema results in a deep, unmodulated voice [3,4]. Reinke’s oedema causes the voice to become hoarse, which causes patients to complain of vocal problems [2,5].

Smoking is considered the main risk factor for Reinke’s oedema; however, laryngopharyngeal reflux and vocal fold abuse also contribute to the development of this condition. It must be noted that allergies are not considered a crucial factor in the aetiology of Reinke’s oedema [6,7,8,9]. The probability of suffering from Reinke’s oedema is higher in older women (>39 years old), but a predilection in males has also been found by some authors [2,10].

Various morphological changes can be found in Reinke’s oedema, such as subepithelial vascularization, thickened basement membrane, dilated capillaries, dense reticular fibres around the vessels, inflammatory cell infiltration, elongated intracellular junctions, and widening of intracellular spaces [11,12].

Risk factors for Reinke’s oedema can lead to vocal fold injury. The reaction of the vocal fold mucosa due to the damage may be excessive extracellular matrix production. A strict correlation between the nuclear protein Ki-67 and cell proliferation has been found [13]. Proliferation marker Ki-67 (Ki-67) expression is high in proliferating cells and absent in resting cells [14]. Thus, for the determination of the cell growth fraction, Ki-67 is used, and Ki-67 staining intensity is increased from the onset of the S phase until metaphase [15].

In cases of vocal fold trauma, the inflammatory response has been demonstrated to play a major role in the removal of injured tissue and reactions with invading organisms. Wound healing initially starts with the inflammatory stage, in which the recruitment of inflammatory cell infiltration, synthesis of growth factors and cytokines, and blood flow changes occur; this means that inflammatory inhibitors, including cytokines, are very important. B cells, mast cells, eosinophils, macrophages, and many subsets of T cells produce interleukin 10 (IL-10), which is widely known for its anti-inflammatory properties [16,17].

The damage of the tissue can promote the initiation of morphological alterations as well as cytokine release. IL-10 has an essential role in the regulation of immune responses, limiting the host immune response, preventing damage, and maintaining homeostasis [18]. Tissue motion can trigger an increase in IL-10 concentration [19]. Interestingly, average normalized IL-10 concentrations have been detected to be the highest 24 h after resonant voice exercises and the lowest after vocal rest [16].

One of the major proinflammatory interleukin-1 (IL-1) family members is interleukin 1 alpha (IL-1α). Two main proinflammatory forms of IL-1 are IL-1α and IL-1β, whose effects are mediated through the IL-1R1 receptor. Healthy individual mesenchymal-originated cells constitutively contain the IL-1α precursor [20,21,22]. The IL-1α precursor is localized in the cytosol and the nucleus, where it is bound to chromatin [23]. The release of IL-1α from macrophages is triggered by necrotic cells in vitro. The blockage of IL-1α influences the acute neutrophilic response but has barely any effects on monocytes. IL-1α necrotizing cells stimulate neutrophil infiltration [24].

Another important immunohistochemical marker is protein gene peptide 9.5 (PGP 9.5), which is also known as ubiquitin C-terminal hydrolase (UCHL-1). PGP 9.5 has demonstrated ubiquitin carboxy-terminal ethyl esterase activity, and PGP 9.5 is known to be widely used as a neural and neuroendocrine cell marker [25,26,27,28].

The aim of this study was to investigate the distribution and appearance of Ki-67, IL-1α, IL-10, and PGP 9.5 in Reinke’s oedema-affected larynx tissue compared with control tissue.

## 2. Materials and Methods

### 2.1. Characteristics of the Subjects

This study was approved by the Ethical Committee of Riga Stradins University. The approval was issued on 31 October 2019 (no. 6-2/2/25). We examined five vocal fold samples from patients with Reinke’s oedema (1 male and 4 females) aged 58 to 71 years (all were irregular smokers). The control group included 2 males and 5 females aged 50 to 75 years. We evaluated seven larynx tissue samples that were obtained from the control group during postmortem autopsies. Control tissue samples were obtained approximately at the same time as the patient tissue. All samples were stained simultaneously. The fixation and embedding method were the same. The control samples used for this study were the property of the Institute for Anatomy and Anthropology of Riga Stradins University.

### 2.2. Routine Histological Analysis

The biopsy samples were fixed for 24 h with 2% formaldehyde and 0.2% picric acid in 0.1 M phosphate (pH = 7.2). After fixation, Tyrode’s buffer was applied to biopsy tissue for 12 h and then embedded into paraffin. Four-micrometre-thick sections were cut and stained with haematoxylin and eosin. This staining was used to evaluate tissue morphological structure.

### 2.3. Immunohistochemical Analysis

For the detection of Ki-67, PGP 9.5, IL-10, and IL-1α, the biotin–streptavidin biochemical method was used. Rabbit antibodies were used for the detection of Ki-67 (CMC27531040, diluted 1:100, Sigma-Aldrich, St. Louis, MO, USA), IL-10 (orb100193, diluted 1:600, Biorbyt, Ltd., Cambridge, UK), PGP 9.5 (439273A, diluted 1:100, Zymed Laboratories, Invitrogen Corporation, Carlsbad, CA, USA), and IL-1α (orb308737, diluted 1:100, Biorbyt Ltd.).

The evaluation of immunoreactive cells in larynx tissue in ten random visual fields at ×400 (ocular ×10, objective ×40) was performed. Ki-67 immunoreactive cells were counted in ten randomly selected visual fields at ×400. In order to avoid subjectivity, the evaluation was performed by two researchers. For further data analysis, the median value was used. The slides were examined under a light microscope (Leica DC 300F, Leica Biosystems Richmond, Richmond, VA, USA). Image-Pro Plus 6.0 software (Media Cybernetics, Inc., Rockville, MD, USA) was used for image analysis.

### 2.4. Quantification of Immunoreactive Cells

To evaluate of the appearance and local distribution of IL-10, IL-1α, and PGP 9.5 immunoreactive structures, a semiquantitative grading method was used [28,29,30]. The semiquantitative scoring system was labelled as follows: 0, negative staining (0%); 0/+, occasional positive structures (12.5%); +, few positive structures (25%); +/++, few to a moderate number of positive structures (37.5%); ++, moderate number of positive structures (50%); ++/+++, moderate to numerous positive structures (62.5%); +++, numerous positive structures (75%); +++/++++, numerous to abundant positive structures (87.5%); ++++, an abundance of positive structures (up to 100%) in the visual field [31].

### 2.5. Statistical Analysis

For statistical analysis of the data, nonparametric statistical methods were used. For the analysis of the IL-10-, IL-1α-, PGP 9.5-, and Ki-67-positive structures within the larynx samples from patients with Reinke’s oedema and controls, the Mann–Whitney U test was used [30,32].

The collected data were transformed to SPSS as follows: negative staining, 0; occasional stained structures, 0.5; few stained structures, 1.0; few to moderate number of stained structures, 1.5; moderate number of stained structures, 2.0; moderate to numerous stained structures, 2.5; numerous stained structures, 3.0; numerous to abundant stained structures, 3.5; and abundant stained structures, 4.0.

Spearman’s rank correlation coefficient rs (Spearman’s rho) was calculated for the evaluation of the cross-compliance of two variables [33]. The acquired correlation coefficient (rs) results were interpreted as follows: 0.80–1.00, very high; 0.60–0.80, high; 0.40–0.60, moderate; 0.20–0.40, low; and <0.2 very low correlation. Statistically significant values were considered *p* values of ≤0.05. Statistical analysis of the data was performed using the statistical software SPSS v22.0 (IBM Co., North Castle, Armonk, NY, USA).

## 3. Results

### 3.1. Findings of Routine Histological Analysis

**Figure 1:**
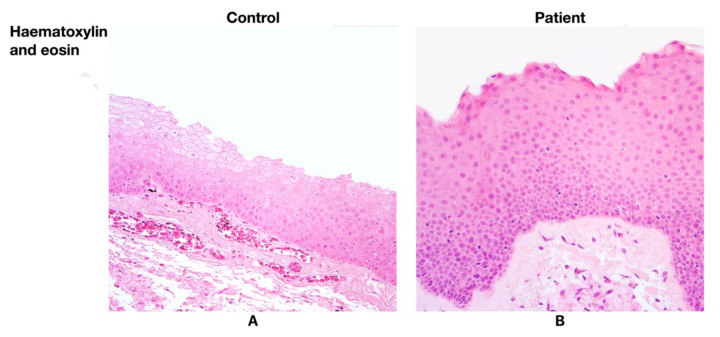
(**A**) No morphological changes were found in larynx mucosa of the control group. Haematoxylin and eosin, ×200. (**B**) Note the basal cell hyperplasia and the thickening of basal membrane in the patient group. Haematoxylin and eosin, ×200.

Having examined the larynx tissue from the patients with Reinke’s oedema, various degrees of basal cell hyperplasia and thickening of the basement membrane were found. Intraepithelial infiltration was detected in three patient samples, while the infiltration in the control group was not predominant (Figure 1A,B).

### 3.2. Immunohistochemistry Findings

**Figure 2:**
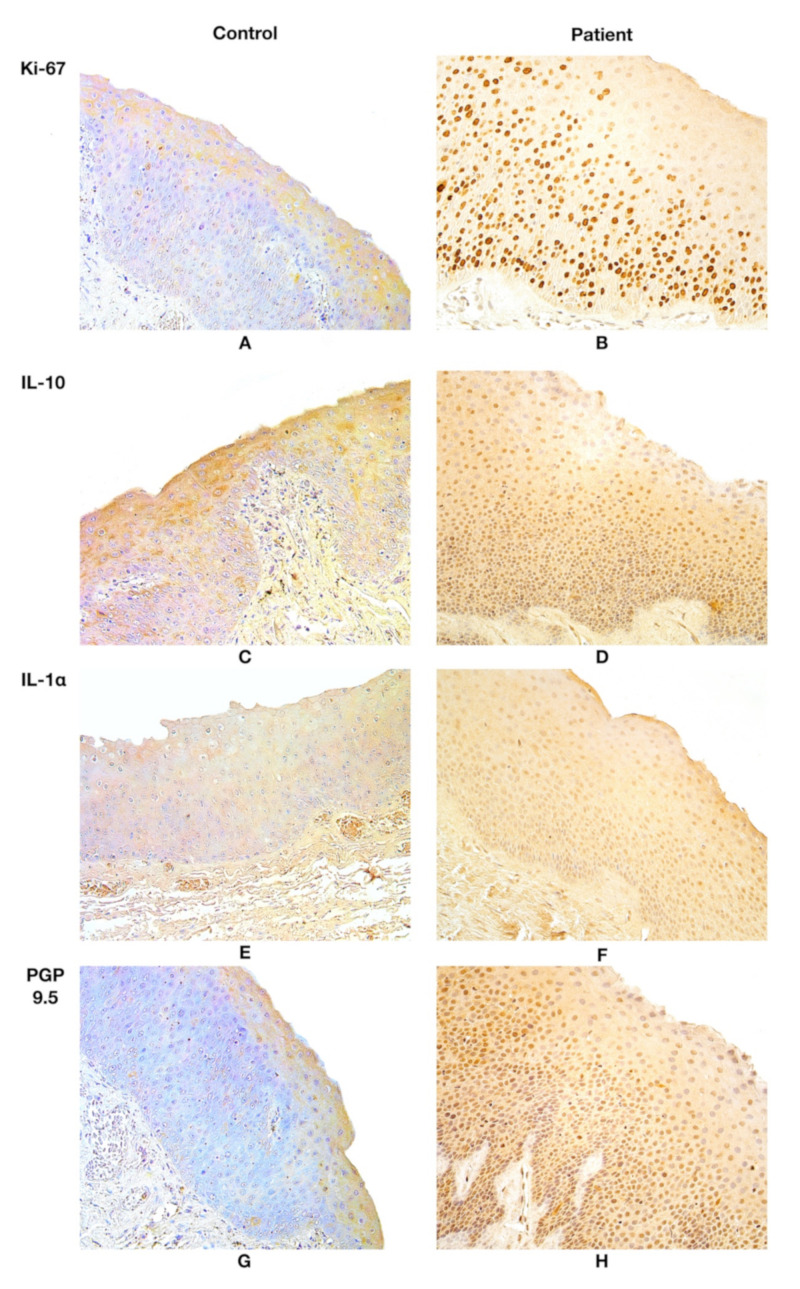
(**A**) Almost no positive Ki-67 structures were found in the control group. Ki-67, ×200. (**B**) Note Ki-67 positive cells in the patient group. Ki-67, ×200. (**C**) Note 0/+ positive number of IL-10 positive structures in the epithelium and subepithelium of the control group. IL-10, ×200. (**D**) IL-10 marks ++/+++ immunoreactive epithelial and subepithelial cells in the patient. IL-10; ×200. (**E**) 0/+ positive IL-1*α* epithelial and subepithelial structures in the control. IL-1*α*, ×200. (**F**) ++/+++ IL-1*α* immunoreactive structures in the epithelium and subepithelium of the patient. IL-1*α*, ×200. (**G**) 0/+ PGP 9.5 epithelial NEC and PGP 9.5 subepithelial nerve appearance in the control group. PGP 9.5, ×200. (**H**) +/++ number of PGP 9.5 positive epithelial NEC and + positive subepithelial nerves in the patient group. PGP 9.5, ×200. Abbreviations: Ki-67—proliferation marker, IL-10—interleukin 10, IL-1α—interleukin 1 alpha, PGP 9.5—protein gene peptide 9.5; NEC—neuroendocrine cells. 0/+—occasional positive structures, +—few positive structures, +/++—few to a moderate number of positive structures, ++/+++—moderate to numerous positive structures.

In all patient samples, Ki-67-positive structures were observed. Ki-67 displayed a median value of 140.5 positive epithelial cells in the patient group, but in the control group, Ki-67 was absent (Table 1, Figure 2A,B). 

IL-10 presented a stable expression of moderate to numerous positive structures both in the epithelium and the subepithelium in the patient group tissues. IL-10 was almost absent in the epithelium and the subepithelium of the control group tissues, marking occasional immunoreactive cells (Table 1, Figure 2C,D).

The patient group presented a high fluctuation in epithelial IL-1α expression, observing few to abundant IL-1α positive structures in visual fields. Moderate to numerous IL-1α positive structures were noted both in the epithelium and the subepithelium in the patient group; however, the control group demonstrated occasional findings (Table 1, Figure 2E,F). IL-10 and IL-1α were expressed in Reinke’s oedema-affected larynx epithelium and subepithelium equally, showing moderate to numerous immunoreactive cells. 

Although the median value for PGP 9.5 expression in epithelial neuroendocrine cells (NEC) of the patient group was few to moderate positive structures, high fluctuations in expression were noted, in which the expression differed from occasional positive to numerous positive structures. Meanwhile, the PGP 9.5 expression in epithelial NEC tissue of the control group showed stable expression across all subjects, showing occasional positive immunoreactive structures. A notable increase in PGP 9.5 was noted in the epithelial tissue, whereas subepithelial tissue showed fewer positive structures of PGP 9.5 subepithelial nerves in the patient group; however, controls revealed a stable number of occasional PGP 9.5 immunoreactive structures, epithelial as well as subepithelial. (Table 1, Figure 2G,H). More detailed information about findings of the semiquantitative eval-uation is available in the Supplementary Material Tables S1–S4.

### 3.3. Statistical Analyses

The following statistically significant differences were found between the patient and control groups: Ki-67 epitheliocytes (Mann–Whitney U: 0; Z-score: −3.169; *p*-value: 0.002), IL-10 epitheliocytes (Mann–Whitney U: 0; Z-score: −3.004; *p*-value: 0.003), IL-10 subepithelial cells (Mann–Whitney U: 0; Z-score: −3.317; *p*-value: 0.001), IL-1α epitheliocytes (Mann–Whitney U: 0; Z-score: −3.004; *p*-value: 0.003), IL-1α-positive subepithelial cells (Mann–Whitney U: 0; Z-score: −3.24; *p*-value: 0.001), PGP 9.5-positive epithelial NEC (Mann–Whitney U: 0; Z-score: −2.952; *p*-value: 0.003), and PGP 9.5 positive subepithelial nerves (Mann–Whitney U: 0; Z-score: −3.064; *p*-value: 0.002).

Spearman’s rank coefficient revealed a low positive correlation between IL-1α epithelial and IL-1α subepithelial immunoreactive cells in the patient group [rs = 0.285; *p* = 0.045]. 

## 4. Discussion

In this study, the comparison of immunoreactive structures between the patient and the control group was performed to determine the significance of Ki-67, IL-10, IL-1α, and PGP 9.5 in the development of Reinke’s oedema. All examined markers in Reinke’s oedema-affected larynx tissue revealed statically significant (*p* < 0.05) changes between the patients and the controls, with a higher number of immunoreactive positive structures in the patient tissue. 

Of all examined markers, only Ki-67 showed no expression in the epithelium of the control tissue. We assume that the Ki-67 expression differences between the patients and controls may be explained by proliferative changes. The increased Ki-67 expression findings in patients reflect an active proliferative process, which could be caused by continuous inflammation in larynx tissue due to persistent risk factors, for instance, smoking. Reinke’s oedema-affected larynx tissue has higher proliferative activity than epithelial cells without oedema [4]. It should be noted that Ki-67 requires an active cell division cycle for its expression, explaining the absence of Ki-67 in the controls, as they were obtained during a postmortem autopsy. A reduction and lack of Ki-67 immunoreactive cells have been found in postmortem material, as proliferation does not occur [13,14,15,34,35].

Statically significantly higher epithelial and subepithelial IL-10 expression in patients in comparison with the controls suggests that IL-10 plays an important role in the development of Reinke’s oedema. Not only IL-1α but also IL-10 is involved in the regulation of neutrophil infiltration during the inflammatory process. We assume that IL-10 expression is related to the limitation of tissue damage due to anti-inflammatory properties by suppressing proinflammatory cytokines, while elevated IL-1α expression indicates inflammation in patients. Additionally, IL-10 expression is related to the phonation process, as the tissue motion triggers IL-10 expression. These aspects may explain the higher IL-10 expression in our patients. Notably, prolonged phonation could damage the vocal folds, stimulating inflammation and IL-1α expression, thus indirectly repeatedly stimulating IL-10 expression [17,18,19,24].

Reinke’s oedema-affected larynx epithelium and subepithelium revealed a higher expression of IL-1α than those in the control tissue samples. We suggest that proinflammatory cytokine IL-1α expression in patients could be higher because of cellular stress-associated factors as well as cell damage. A wide spectrum of possible stimuli could promote these expression changes; for instance, a direct effect of airflow on the larynx, microbial contamination, inhaled particles, and extensive vocal exercise may constitutively increase IL-1α expression, thus promoting inflammation. A higher intensity of phonation has caused increased expression of inflammatory mRNA in normal rabbit vocal folds [36]. Additionally, during routine histological examination, intraepithelial infiltration was noted in the samples of the patient group. The patient group presented a low positive correlation between IL-1α epithelial and IL-1α subepithelial immunoreactive cells, suggesting that changes in the epithelium and subepithelium of Reinke’s oedema-affected larynxes are interacting.

This observation supports the concept that IL-1α release might be related to cell injury. In accordance with other authors, theoretically, this cytokine may be used as an indicator for tissue health. Notably, elevated levels of IL-1α expression are also related to epithelial proliferation, loss of cell attachment, and cellular apical migration. It could modulate larynx tissue proliferation and remodelling processes. IL-1α is released from cells because of cell death or injury. We hypothesized that increased IL-1α in Reinke’s oedema is connected to persistent inflammation, proliferation, and tissue remodulation [37,38].

Reinke’s oedema-affected larynx tissue revealed significantly higher PGP 9.5 epithelial NEC as well as subepithelial expression in the patient group. Studies have shown that excess neuropeptides lead to an increase in immune infiltrates, which can promote the remodelling process. Therefore, we suggest that increased PGP 9.5 expression of patients could be a result of persistent inflammation and chronic irritation of environmental factors, which was also observed by other scientists [39].

Finally, we presume that Ki-67, IL-10, IL-1α, and PGP 9.5 play a role in the developmental process of the morphopathogenesis in Reinke’s oedema. These immunoreactive markers could be involved in inflammation, proliferation, and remodelling of the larynx.

## 5. Conclusions

Reinke’s oedema-affected larynx tissue shows intensive proliferation of the surface epithelium; this could be due to persistent environmental factors (smoking) and inflammation. The notable increase in IL-10-positive structures indicates the dominant anti-inflammatory tissue response.

An increased number of IL-1α structures in the larynx epithelium and subepithelium in the patient group is linked to inflammation, proliferation, and tissue remodelling. PGP 9.5 expression increase is involved in the morphopathogenesis of Reinke’s oedema.

## Figures and Tables

**Table 1:** Median number of Ki-67 positive cells and median values of the semiquantitative evaluation IL-10, IL-1α, PGP 9.5 in the patient, and control groups.

Factors/Subjects	Ki-67	IL-10		IL-1α		PGP 9.5	
	Epithelial	Epithelial	Subepithelial	Epithelial	Subepithelial	Epithelial NEC	Subepithelial Nerves
P1	229.0	++/+++	++/+++	++	++/+++	+/++	+
P2	140.5	++/+++	++/+++	++/+++	++/+++	+/++	+/++
P3	139.0	++/+++/+++	++/+++	++/+++	++/+++	+/++	+
P4	204.5	++/+++	++/+++	++/+++	++/++/+++	+/+/++	+
P5	126.0	++/+++	++/+++	++/+++	++/+++	+/++	+/++
Median (P)	140.5	++/+++	++/+++	++/+++	++/+++	+/++	+
IQR (P)	84.25	0.125	0.00	0.25	0.125	0.125	0.5
C1	0.00	0/+	0/+	0/+	0/+	0	0/+/+
C2	0.00	0/+	0/+	0/0/+	0/+	0/+	0/+
C3	0.00	0/+	0/+	0/+	0/+	0	0/+
C4	0.00	0/+	0/+	0	0/+	0/+	0/+
C5	0.00	0/0/+	0/+	0/+	0/+	0/0/+	0/+
C6	0.00	0/+	0/+	0/+	0/+	0/+	0/+
C7	0.00	+	0/+	0/+	0/+	0/+	0/+
Median (C)	0.00	0/+	0/+	0/+	0/+	0/+	0/+
IQR (C)	0.00	0.00	0.00	0.25	0.00	0.5	0.00

Abbreviations: Ki-67—proliferation marker, IL-10 —interleukin 10, IL-1α —interleukin 1 alpha, PGP 9.5 —protein gene peptide 9.5, NEC —neuroendocrine cells. P1–P5—median value in each patient sample, C1–C7—median value in each control sample, Median (P)—median value in the patient group sample, Median (C)—median value in the control group sample, IQR (P)—interquartile range in the patient group sample, IQR (C)—interquartile range in the control group sample, 0—negative staining, 0/0/+—between no positive structures and occasional positive structures, 0/+—occasional positive structures, 0/+/+—between occasional positive structures and few positive structures, +—few positive structures, +/+/++—between few positive structures and few to a moderate number of positive structures, +/++—few to a moderate number of positive structures, ++—moderate number of positive structures, ++/++/+++—between a moderate number of structures and moderate to numerous positive structures, ++/+++—moderate to numerous positive structures, ++/+++/+++—between moderate to numerous positive structures and numerous positive structures in the visual field.

## Data Availability

All data generated or analysed during this study are included in this published article.

## Supplementary Materials

The following are available online at https://www.mdpi.com/article/10.3390/life11121379/s1, Table S1: Semiquantitative evaluation of immunoreactivity of PGP 9.5; IL-1α; IL-10 patient and control groups in 10 visual fields; Table S2: Median values of semiquantitative evaluation of immunoreac-tivity of PGP 9.5; IL-1α; IL-10 in patient and control groups; Table S3: Ki-67 positive cells in one visual field in patient and control groups; Table S4: Median number of Ki-67 positive cells in patient and control groups.
